# Perinatal and Delivery Outcomes Following Amniocentesis: A Case-Control Study in the Polish Population

**DOI:** 10.3390/jcm14020309

**Published:** 2025-01-07

**Authors:** Daniel Wolder, Anna Blazuk-Fortak, Agata Michalska, Karolina Detka, Grzegorz Świercz, Piotr Kaczmarek

**Affiliations:** 1 Department of Medicine, Jan Kochanowski University, 25-369 Kielce, Poland; ab.ab.blazuk@gmail.com (A.B.-F.); karolina.cedro@poczta.fm (K.D.); grzegorz.swiercz60@gmail.com (G.Ś.); 2 Institute of Health Sciences, Collegium Medicum, The Jan Kochanowski University, 25-516 Kielce, Poland; michalska.agata@ujk.edu.pl; 3 Polish Mother’s Memorial Hospital Research Institute, 93-338 Lodz, Poland; kaczmarekpiotr1@gmail.com

**Keywords:** prenatal care, genetic counseling, prenatal diagnosis, prenatal screening

## Abstract

**Background:** Amniocentesis is a widely used invasive prenatal diagnostic procedure, recognized for its high sensitivity and low risk of complications. This study aims to evaluate the association between amniocentesis and pregnancy outcomes, such as miscarriage, preterm rupture of membranes (PROM), and preterm birth, as well as perinatal outcomes. **Methods:** A case-control study was conducted at the Regional Hospital in Kielce, Poland, from 2016 to 2022, involving 1834 patients, 225 of whom underwent amniocentesis, while 1609 did not receive any invasive diagnostics. Data were collected from medical records and included maternal factors such as age, BMI, delivery mode, complications, and newborn condition. **Results:** The study found no statistically significant differences between the study and the control groups regarding pregnancy or perinatal characteristics. Miscarriage occurred in 1.9% of the patients in the amniocentesis group, with no cases in the control group. Rates of preterm delivery were similar between groups (8.33% in the study group vs. 5.74% in the control group, *p* > 0.05). Postnatal outcomes, such as birth term, birth weight, and Apgar scores, were comparable across both groups. Fetal growth restriction was slightly more frequent in the study group (2.8% vs. 0.8%). One neonatal death was observed in each group. The relative risk of complications following amniocentesis was 1.69 (CI 0.38–7.24). **Conclusions:** Amniocentesis is a safe invasive prenatal procedure. It should be offered to every pregnant woman when necessary. Before the procedure, the patient should be clearly informed of the risk related to amniocentesis but at the same time reassured that the complication rate is very low.

## 1. Introduction

Amniocentesis is an invasive prenatal diagnostic tool which has seen the fastest development in recent years, making it one of the leading diagnostic tests. This procedure, followed by genetic testing, is regarded as the “gold standard” of invasive prenatal procedures owing to its low complication risk and near-perfect sensitivity, approaching 100 percent [1]. First introduced in clinical trials in the 1970s, amniocentesis involves the sampling of amniotic fluid to isolate amniocytes. Culturing these amniocytes allows for the assessment of the fetal karyotype, which remains the most common method of prenatal genetic testing. However, the use of microarray comparative genomic hybridization (aCGH) is becoming increasingly popular [2]. Cytogenetic testing encompasses the detection of chromosomal aberrations, such as aneuploidies, translocations, duplications, and deletions. Invasive prenatal diagnostics are indicated in several scenarios: when non-invasive screening tests suggest a high risk (> 1:300) of chromosomal aberrations, when the presence of anatomical defects is detected via ultrasound, following abnormal non-invasive prenatal testing (NIPT) results, when the patient has an abnormal genetic history, or when there is suspicion of intrauterine infections [3].

The risk of complications associated with amniocentesis is estimated to range from 0.1% to 1% [4,5,6]. The risk of miscarriage after amniocentesis increases proportionally to the mother’s age, the number of insertions, the presence of cancerous tumors, and the mother’s BMI, while it is inversely proportional to the operator’s experience [7]. Fetal and maternal complications can arise at any stage of a pregnancy; therefore, identifying potentially influential factors with respect to maternal and fetal health and assessing the outcomes of amniocentesis is crucial for prenatal care, especially when the risk of fetal genetic anomalies is high.

This study aims to evaluate the association between amniocentesis and pregnancy outcomes as well as perinatal results. The complications assessed include miscarriage, preterm rupture of membranes (both preterm PROM and PROM), and preterm delivery. The perinatal outcomes analyzed comprise the term of birth, neonates’ weight, neonates’ general condition assessed using the Apgar scale, and instances of neonatal death.

## 2. Materials and Methods

### 2.1. Study Design

It is a case-control study including a group of 1834 patients (225 patients qualified for amniocentesis and 1609 patients did not undergo any invasive prenatal diagnostic test). This study was conducted in accordance with the STROBE [8] (Strengthening the Reporting of Observational Studies in Epidemiology) guidelines.

### 2.2. Setting

The study was conducted in the Regional Hospital in Kielce, Poland, in the period between 2016–2022. In this healthcare setting, amniocentesis is fully reimbursed through public health funding, whereas NIPT is not covered and requires out-of-pocket payment from patients. This financial aspect significantly influences the choice of diagnostic method, with many patients opting for the publicly funded procedure. Regarding CVS, while it is a valuable diagnostic tool, it is a technically more demanding procedure that requires specialized training and experience. At this center, no specialists were available with the qualifications required to perform CVS during the study period. As a result, amniocentesis was the preferred option for invasive prenatal testing.

### 2.3. Participants

The inclusion criteria for this study were: a screening prenatal test conducted between 11 and 13 + 6 weeks of pregnancy, performed as indicated by the Polish Society of Gynecology and Obstetrics according to standardized rules [9], access to the results of the karyotype test following amniocentesis, and access to data concerning pregnancy and delivery outcomes. Exclusion criteria included the absence of data concerning pregnancy and delivery outcomes, the absence of karyotype test results following amniocentesis, and cases of multiple pregnancies.

### 2.4. Variables and Data Sources

The data were sourced from the electronic medical records of pregnant women, their hospital stay during delivery, and their newborns. Detailed variables collected from medical histories included the mother’s age, parity, BMI, gestational age, mode of delivery, number of hospitalizations, maternal anemia, herpes infection during pregnancy, COVID-19 infection during pregnancy, presence of group B streptococcus (GBS), and chronic diseases such as diabetes, hypertension, hypothyroidism, and thrombocytopenia. Additionally, the occurrence of respiratory and urinary tract infections during pregnancy, a history of antiphospholipid syndrome, occurrence of systemic lupus erythematosus, Hashimoto’s disease, smoking, and breech presentation during delivery were recorded.

Complications during pregnancy and delivery were also assessed, including vaginal bleeding, preterm labor, the premature rupture of membranes, threatened preterm labor, cervical pessary placement, miscarriage, polyhydramnios, oligohydramnios, anhydramnios, intrahepatic cholestasis of pregnancy, mode of delivery completion, and labor induction. The karyotype of the study group was evaluated as well.

The condition of the newborn was assessed after three minutes of life using the Virginia Apgar scale. Furthermore, the newborn’s birth weight in grams and the occurrence of fetal growth restriction (FGR) were evaluated. FGR was determined based on Hadlock percentile charts with the cutoff point at the third percentile. The occurrence of neonatal deaths was also recorded.

### 2.5. Study Size

Due to the significant disparity in the number of patients between the control and study groups, the control group patients were selected randomly. From the control group, patients were randomly selected and matched based on age (±1 year), body mass index (BMI) and body weight (±2 kg), and parity. Following the final analysis of inclusion and exclusion criteria, 231 patients were qualified for the study: 109 who had undergone invasive prenatal diagnostics and 122 who served as the control group.

### 2.6. Procedures

The integrated test comprised an ultrasound examination conducted between the 11th week and 13th week plus 6 days of pregnancy, along with a biochemical assessment of genetic markers, specifically the PAPP-A test and the beta HCG. The ultrasound examinations were performed in accordance with the recommendations of the Polish Society of Gynecologists and Obstetricians (PTGiP) by certified professionals holding credentials such as the FMF certificate and the PTGiP prenatal testing certificate. The ultrasound equipment used allowed for real-time 2D imaging in various shades of gray and enabled measurements of distance, circumference, and area. It also featured Doppler options, including color and pulsed Doppler, and was equipped with both transabdominal and transvaginal probes, enabling image capture and electronic documentation. In the Provincial Integrated Hospital in Kielce, laboratory tests were conducted by the external company Diagnostyka Laboratoryjna using the KRYPTOR device, which is the reference equipment recommended by the Fetal Medicine Foundation (FMF).

The laboratory tests involved the assessment of two protein markers, PAPP-A and ß-hCG, using the KRYPTOR device. To accurately calculate the individual risk for a specific pregnant woman, it was necessary to consider appropriate adjustments for the measurement of free ß-hCG and PAPP-A. Each measured value was expressed as a multiple of the median (MoM) of normal values for the given gestational age, adjusted for the pregnant woman’s body weight, smoking status, ethnic origin, and method of conception.

The ultrasound examination included the assessment of genetic markers such as the nuchal translucency (NT) measurement, the fetal heart rate (FHR) evaluation, the crown-rump length (CRL) measurement, and the evaluation of additional ultrasound markers including the presence of the nasal bone (NB), ductus venosus flow (DV-PI), and tricuspid valve flow (TR). Amniocentesis was performed under ultrasound control after 15 weeks of pregnancy with the use of a G20 needle that enabled collection of 15 mL of amniotic fluid. The chosen genetic test was microarray comparative genomic hybridization. While performing amniocentesis, the aim was to avoid going through the placenta.

### 2.7. Statistical Analysis

The RStudio software was used for statistical analyses. Quantitative data characterized by a normal distribution were presented as their mean value and standard deviation, whereas quality data were presented as the number of cases and the corresponding percentage. A student’s *t*-test was selected to evaluate the difference among quantitative variables, while a Pearson’s chi-squared test was chosen for quality data comparison. A *p*-value of <0.05 was considered to be statistically significant.

### 2.8. Ethical Considerations

The study was approved by the Bioethical Committee of the Jan Kochanowski University in Kielce (no. 49/2022; date: 10 November 2022).

## 3. Results

### 3.1. Groups Characteristic

Selected pregnancy and perinatal characteristics (Table 1) were used to compare both groups in the study. No statistically significant differences were observed between the groups.

After performing genetic tests following amniocentesis, it was observed that the karyotype in the majority of cases was normal (Table 2). There were more female fetuses with trisomy 21, whereas the number of fetuses with trisomy 18 of both sexes was the same. Other chromosomal aberrations were less common. In addition, there was no fetus with trisomy 13 in the study group.

### 3.2. Prenatal Complications

**Figure 1:**
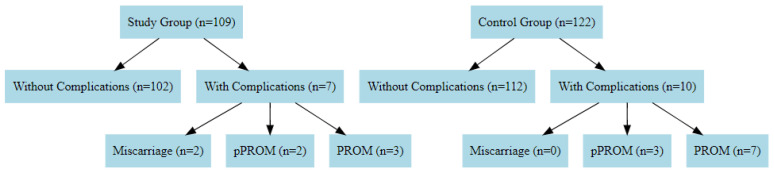
Flowchart of pregnancies and prenatal complications in the study and control groups.

Two patients (1.9%) from the study group had a miscarriage. The karyotype results of both fetuses were normal. The first miscarriage happened in the 17th week of pregnancy, approximately fourteen days after the amniocentesis. The specific data concerning the second case could not be collected. In the control group, there was no miscarriage reported. There were two cases (2%) of pPROM reported in the study group and three cases (2.5%) in the control group. The first case in the study group occurred approximately four weeks after the amniocentesis, and the second in the 33rd week of pregnancy. The premature rupture of membranes was observed in three patients from the study group (3%) and in seven (5.7%) in the control group (Figure 1). Preterm delivery (< 37 weeks of pregnancy) occurred in 8.33% of the women in the study group and in 5.74% of the women in the control group. No statistically significant differences were observed with respect to any of these variables.

**Figure 2:**
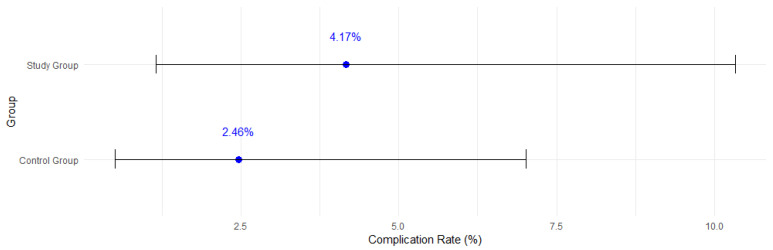
Composite outcome rate analysis in the study and control group, *p* = 0.447.

An analysis of the composite outcomes (miscarriage and pPROM) was performed in both groups. Four adverse events in the study group were reported, in comparison to three in the control group (Figure 2). That difference was not significant. The relative risk (RR) of complications following amniocentesis in the study group equaled 1.69 (CI 0.38–7.24).

### 3.3. Postnatal Outcomes

Statistically significant differences in the term of birth and birth weight between the two groups were not noted in the study (Table 3). There were also no statistically significant differences in gender distribution between the groups, with females accounting for 37.20% of the study group and 49.20% of the control group, and males accounting for 62.80% of the study group and 50.80% of the control group (*p* = 0.106).

The condition at birth of most of the neonates in both groups was assessed as optimal. That means that they obtained 10 points in the Apgar scale (83% of the neonates in the study group and 82% in the control group) (*p* = 0.757). FGR was observed in 2.8% of the neonates from the study group in comparison to 0.8% of neonates from the control group (*p* = 0.517). One case (1.04%) of neonatal death was reported in the study group (neonate born after 23 weeks of pregnancy) and one case (0.82%) of neonatal death was observed in the control group (neonate born after 25 weeks of pregnancy) (*p* = 1.000).

## 4. Discussion

### 4.1. Main Results

The principal finding of this study is that amniocentesis is a procedure with a very low risk of pregnancy and perinatal complications. A higher prevalence of complications, such as miscarriage, pPROM, PROM, or preterm delivery, was not observed. Moreover, there was no significant difference in perinatal results, including birth term, birth weight, and general neonates’ condition, at birth and no significant difference in neonatal death between the two groups.

### 4.2. Reference to Other Research

The results of this study align with findings from other research on amniocentesis and its associated maternal–fetal complications. A review by Jummaat et al. examined various factors, including maternal age, indications for amniocentesis, gestational age at the time of the procedure, karyotype results, and complications during or after the procedure. Their findings indicate that amniocentesis was a safe procedure for 86% of their patients [10]. 

In our study, the premature rupture of membranes occurred in 0.9% of the patients, a value which is consistent with the 2.6% rate reported by Jummaat et al. [10] and with findings from other studies, where the incidence ranges from 1% to 2% [11,12].

Preterm labor, defined as the delivery before 37 weeks, occurred in 8.33% of the patients in the study group compared to 5.74% of the patients in the control group, with no statistically significant difference (*p* = 0.615). Wen-Wei Hsu et al. reported similar results, with preterm delivery rates of 9.4% in the amniocentesis group and 7.5% in the control group (*p* < 0.001) [13]. Their study included a large sample size, with 250,566 patients in the amniocentesis group and 1,134,403 in the control group. Additionally, they analyzed preterm births before 24 weeks, with rates of 0.68% and 0.46% in the two groups, respectively (*p* < 0.001), resulting in an unintended miscarriage rate of 0.22% (0.68–0.46%) [13].

In our study, the miscarriage rate was 1.9% in the study group, while no miscarriages were observed in the control group. One of the most concerning factors during the procedure is transplacental amniocentesis, which can lead to placental or intra-amniotic hemorrhage, fetal placental ischemia, and chorionic vessel damage due to intra-placental hematomas [14,15]. While many studies have reported a slightly increased risk of miscarriage, none have demonstrated statistically significant results [16,17].

Other retrospective studies also show that amniocentesis is not associated with major complications, in most cases. Theodora et al. estimated the risk of fetal loss following second-trimester amniocentesis to be 1.19%, similar to the general procedural risk [18]. Additionally, the premature rupture of membranes was noted in 2% of the patients in the study group and in 2.5% of the patients in the control group, with no statistically significant difference. This is consistent with the findings of Jummaat et al. [13]

### 4.3. Study Limitations

This study has certain limitations. First, there were many patients whose data were incomplete or lost during the process. Additionally, the study does not report on other pregnancy complications, such as vaginal bleeding or intrauterine infections. Finally, the strength of the study group is limited, which may have affected the overall findings. A notable limitation of this study is the small number of preterm deliveries (<37 weeks) in the study population, with fewer than 10 cases per group. This limited sample size did not allow for a separate preterm and term delivery analysis. Future studies with larger cohorts are needed to enable a more detailed assessment of pregnancy outcomes in these subgroups. Another limitation of this study is the presence of several cases of fetal aneuploidy in the study group, which could have potentially confounded the results by worsening perinatal outcomes; however, due to the lack of differences between the study and control groups, this factor does not appear to be applicable.

### 4.4. Clinical Practice

While planning prenatal diagnostic tests, it is crucial to properly assess a patient’s clinical situation and possibilities. Among the indications of amniocentesis, an abnormal NIPT result is the one particularly worth discussing. When choosing between CVS or amniocentesis, it is necessary to remember confined placental mosaicism. In that case, amniocentesis could appear to be a more appropriate choice [19]. The current trend is to focus on non-invasive genetic testing, which means performing NIPT more often. Despite its high sensitivity in detecting most common chromosomal aberrations (trisomy 21, 18, 13), it cannot replace invasive procedures in cases of high-risk genetic malformations [20].

Amniocentesis should be performed after 15 weeks of pregnancy (optimally between 15 weeks and 18 weeks). It has been proven that early amniocentesis (before 15 weeks of pregnancy) might be associated with a greater risk of complications, as well as unsuccessful isolated cell growth [21]. On the other hand, late amniocentesis is associated with a higher risk of culture failure [22]. Medical professionals are advised to inform the patients of the complications following amniocentesis. This invasive procedure should be taken care of differently in instances of multiple pregnancies. It has been stated that its risk is greater in comparison to singleton pregnancies [23]. It is necessary to explain that to the patient. It should be noted that, for certain patients, the decision to undergo amniocentesis is difficult, even after acknowledging its low risk. Women older than 30 years old are more likely to agree to invasive prenatal diagnosis. When asked, the age is said to be the most important factor when making that decision, even without information about genetic malformation risk. On the other hand, the fear of fetal injury is still the main cause behind the decision to decline amniocentesis [24]. Amniocentesis can also allow for the planning of the future course of the pregnancy. For example, the qualification process for fetal myelomeningocele surgery involves reporting a normal fetal karyotype [25]. It is also important that there be no absolute contraindication to amniocentesis. In particular situations such as patients suffering from HIV or hepatitis B infections, oligohydramnios, or undergoing oral anticoagulation therapy, the procedure-related risk should be calculated individually [6].

## 5. Conclusions

Amniocentesis is a safe invasive prenatal procedure. It should be offered to every pregnant woman when necessary. Before the procedure, the patient should be clearly informed about the risk related to amniocentesis but, at the same time, reassured that the complication rate is very low. Clinicians should prioritize individualized patient counseling, particularly after abnormal NIPT results, and consider amniocentesis over CVS in cases of confined placental mosaicism. The optimal timing is between 15 weeks and 18 weeks to minimize risks, with special attention to multiple pregnancies due to their higher complication rates. Addressing patient concerns, especially fears of fetal injury, and explaining the role of amniocentesis in pregnancy planning can support informed decision-making. Clinical scenarios like maternal infections, oligohydramnios, and anticoagulation therapy require individualized risk assessments rather than absolute contraindications.

## Figures and Tables

**Table 1:** Pregnancy and perinatal occurrences characterizing the groups from the study.

Variable	Study Group *N* = 109		Control Group *N* = 122		*p*
	*n*	%	*n*	%	
number of previous deliveries					
0	21	19.3	40	32.8	0.106
1	57	52.3	58	47.5	
2	25	22.9	19	15.6	
3	6	5.5	5	4.1	
Anemia	2	1.9	0	0	0.417
Hypothyroidism	9	8.4	13	10.7	0.726
Antiphospholipid syndrome	1	0.9	1	0.8	1.000
Polyhydroamnion	1	0.9	1	0.8	1.000
Herpes simplex	3	2.8	2	1.6	0.874
Hashimoto	3	2.8	2	1.6	0.874
Thrombosis	0	0	3	2.5	N/A
COVID-19	3	2.8	9	7.4	0.216
GBS +	53	27.3	89	42.1	0.107
GDM	21	19.6	14	11.5	0.127
Low platelet count	1	0.9	2	1.6	1.000
PIH	6	5.6	10	8.2	0.726
Respiratory infection	13	12.3	19	15.6	0.599
UTI	9	8.5	10	8.2	1.000
Pessar	3	2.8	2	1.6	0.874
Fetal breech position	7	6.5	7	5.7	1.000
Placental pathology	2	1.9	3	2.5	1.000
Cholestasis	1	0.9	0	0	N/A
Preeclampsia	0	0	1	0.8	N/A
Mode of delivery					0.156
Vaginal	34	36.2	57	46.7	
C. section	75	63.8	65	53.3	

GBS—group B Streptococcus, GDM—gestational diabetes mellitus, PIH—pregnancy-induced hypertension, UTI—urinary tract infection, N/A—not applicable

**Table 2:** Karyotypes in the study group.

Karyotype	*n* (%)
45X0	1 (0.9)
45XY del 11	1 (0.9)
46XX	35 (32.7)
46XY	57 (53.3)
47XX+18	1 (0.9)
47XX+21	5 (4.7)
47XY+18	1 (0.9)
47XY+21	4 (3.7)
No information	1 (0.9)
Del 21	1 (0.9)

**Table 3:** Birth term and birth weight of the neonates included in the research.

	Study Group M (SD) *N* = 109	Control Group M (SD) *N* = 122	*p*
Birth term (weeks)	38.33 (2.01)	38.58 (1.74)	0.319
Birth weight (g)	3308.97 (571.73)	3370.98 (502.27)	0.398

## Data Availability

The data presented in this study are available on request from the corresponding author.
